# Case report: pulmonary nocardiosis caused by *Nocardia exalbida* in an immunocompetent patient

**DOI:** 10.1186/s12879-021-06416-w

**Published:** 2021-08-09

**Authors:** Seitaro Abe, Yoshinari Tanabe, Takeshi Ota, Fumio Fujimori, Akira Youkou, Masato Makino

**Affiliations:** 1 Department of Respiratory Medicine, Niigata prefecturasl Shibata hospital, 1-2-8 Shibata, Niigata, 957-8588 Japan; 2 Department of Infectious Disease, Niigata City general hospital, Niigata, Japan

**Keywords:** *Nocardia exalbida*, Infection, Pulmonary nocardiosis, Immunocompetent patient

## Abstract

**Background:**

Nocardiosis is known as an opportunistic infection in immunocompromised hosts, but it occasionally has been reported in immunocompetent patient. The *Nocardia exalbida* is first-reported in 2006 from Japan, and a few cases of have been reported in only immunocompromised host, and the characteristic is still unclear. We herein describe the first case of pulmonary nocardiosis caused by *N. exalbida* in an immunocompetent patient.

**Case presentation:**

A77 -year-old Japanese man was admitted to our hospital on November 2, 2018. He was a lifelong non-smoker with no childhood history of respiratory disease. He had a medical history of dyslipidemia. One month before this admission fevers, sputum, mild cough were developed and he was evaluated in a clinic near our hospital. His diagnosis was community acquired pneumonia within his right middle lobe. He was treated with ceftriaxone 1 g/day intravenously for a week, however his symptoms relapsed a few days later. So, the physician retried ceftriaxone for another 3 days, but his symptoms did not improve. He was referred to our hospital. He was treated with sitafloxacin as an outpatient for a week, however his symptoms got worse. The chest CT showed consolidation and atelectasis in his right middle lobe. Low density area was scattered in consolidation, and right pleural effusion was observed.

The patient was diagnosed with pulmonary abscess and he was admitted. Administration of piperacillin/tazobactam improved his condition. We switched antibiotics to amoxicillin/clavulanate, and he was discharged. After 2 weeks, he relapsed and was admitted again. After administration of piperacillin/tazobactam for 3 weeks, we perform bronchoscopy and *Nocardia* species were cultured from samples of the bronchial wash. The isolates were identified as *N. exalbida* using 16S rRNA gene sequencing. We prescribed Trimethoprim / Sulfamethoxazole (TMP/SMX) for 4 months. Then we switched to minocycline for renal dysfunction caused from TMP-SMX for 1 more month. After 5 months therapy, Consolidation on CT disappeared, and Nocardiosis was cured.

**Conclusion:**

we reported the first case of pulmonary nocardiosis caused by *N. exalbida* in an immunocompetent patient. *N. exalbida* infection might be associated with a good response to treatment.

## Back ground

The genus *Nocardia* is an aerobic bacterium, Gram-positive and catalase positive that is in the Nocardiaceae family [1]. Since Edmond Nocard isolated an aerobic filamentous organism from lesions in cattle suffering from fancy in 1888 and Trevisan created the genus *Nocardia* to accommodate Nocard’s isolate and named it *Nocardia farcinica* [1], *Nocardia* species are classified by various methods that have morphological, biochemical, physiological and chemotaxonomic properties.

Of particular interest, the recent introduction of molecular methods, such as sequencing of the 16S rRNA gene, allows for more accurate clarification of the taxonomy of *Nocardia* and new findings are updated. The identification of the *Nocardia* species in nocardiosis is important because the drug susceptibility differs among the species [2].

Nocardiosis is known as an opportunistic infection in immunocompromised hosts, but occasionally it has been reported in immunocompetent patient [3]. The data about worldwide incidence of *Nocardia* infection is limited and unclear. In An American study, Annual incidence of nocardiosis in United State is about 500–1000 cases per year [4]. However some literature points out this incidence was greatly underestimated [5]. Meanwhile, the incidence of nocardiosis may be increasing because of the growing number of immunocompromised patients treated with advances in medical treatment. *Nocardia* infects the lungs, skin, central nervous system (CNS) or other organs presenting as localized or disseminated infections. Pulmonary nocardiosis is the main type of nocardiosis. Nocardiosis involves the lung in 60–70% of cases [6].

We herein report a case of pulmonary nocardiosis caused by *N. exalbida* in a patient who is non-immunocompromised and review the reported cases of *N. exalbida* infection.

## Case presentation

A 77-year-old Japanese man was admitted to our hospital on November 2, 2018. One month before this admission, fevers, sputum, mild cough were developed, and the patient was evaluated in a community clinic. He was a lifelong non-smoker with no childhood history of respiratory disease. A chest x-ray showed consolidation on his right lung field. A clinical diagnosis of right pneumonitis was made. The *α-streptococcus* was isolated from his sputum. He was treated with ceftriaxone intravenously for a week, his symptoms relapsed a few days later. He was re -treated with ceftriaxone for 3 days, his symptoms did not show improvement. The patient was referred to our hospital for management of refractory pneumonitis. His body height was 5′ 2″ (162 cm), his body weight was 63 kg, and his body mass index was 23.6.

**Fig. 1:**
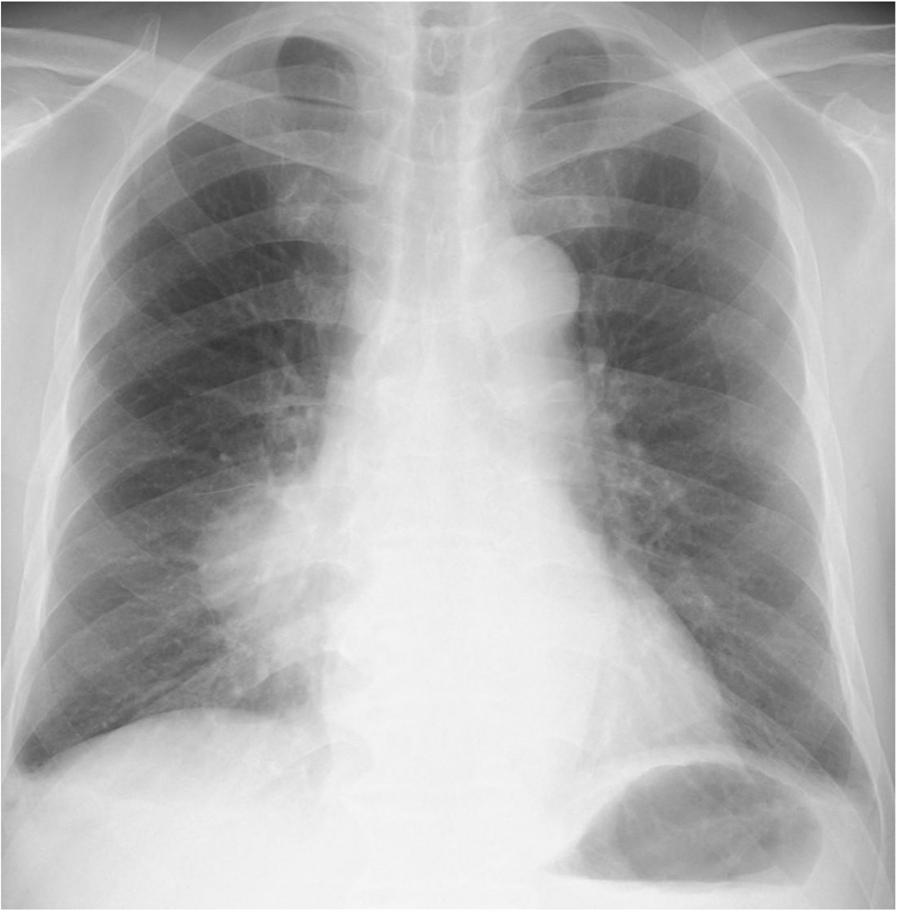
Chest X-ray on 1st admission at the hospital. Consolidation was present in the right lower lung field, and the costophrenic angle was blunted on the right side

**Fig. 2:**
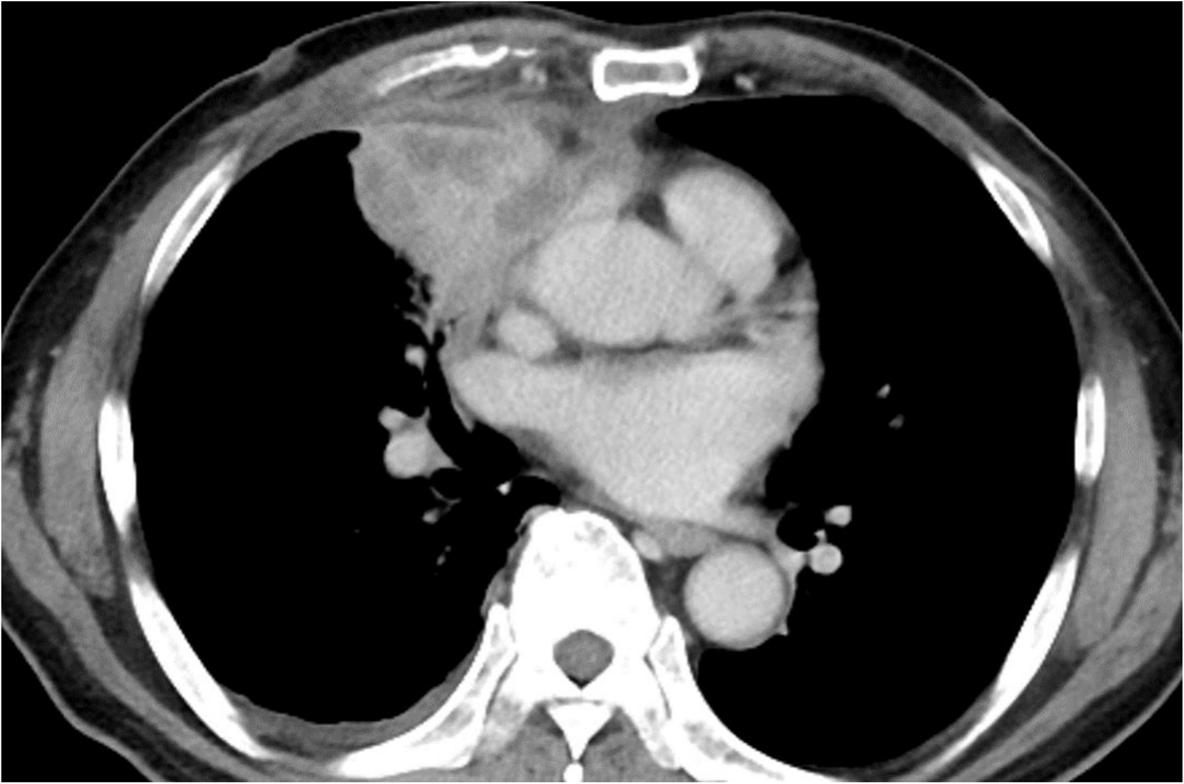
Contrast-enhanced thoracic computed tomography on 1st admission at the hospital. Lung abscess formation was present in right middle lobe. Low density area was scattered in consolidation, and right pleural effusion was present

On physical examination, his consciousness level was clear (GCS; E4V5M6), pulse was 92 beats per minute, respiratory rate was 18 breaths per minute, blood pressure was 119/72 mmHg, temperature was 36.7 °C (98.1 °F), and oxygen saturation was 96% (room air). Auscultation of his lungs revealed that there was decreasing lung sound in his right middle lung field, dermatological findings and neurologic findings were normal. Laboratory evaluation showed an elevated peripheral blood white blood cell count of 13,000 /μL with 88.4% neutrophils, and a C-reactive protein level of 11.29 mg/dL. Baseline investigations for immunodeficiency had normal results. No pathogenic bacteria were detected from a sputum culture. Sputum cytology was class II and neutrophil were abundant. We prescribe sitafloxacin for one more week, however his temperature increased to 38.3 °C (100.9 °F) with right chest pain, bloody sputum, and general malaise. A chest x-ray showed consolidation on his right lung field (Fig. 1). A chest computed tomography (CT) scan showed consolidation and atelectasis in his right middle lobe. Low density area was scattered in consolidation, and right pleural effusion was observed (Fig. 2). He was diagnosed with pulmonary abscess and he was admitted. The laboratory findings on admission (Table 1) demonstrated an elevated peripheral blood white blood cell count (16,100/μL) and a CRP level (17.9 mg/dL).

**Table 1:** Laboratory Findings on Admission

<Blood cell count>		
White blood cell	16,100	/μL
Red blood cell	405	× 10^4^/μL
Hemoglobin	11.9	g/dL
Hematocrit	35.5	%
Platelet	27.4	×10^4^/μL
<Serum chemistry>		
Total Protein	6.6	g/dL
Blood urea nitrogen	18	mg/dL
Creatinine	0.79	mg/dL
Total-bilirubin	1.2	mg/dL
Aspartate transaminase	60	U/L
Alanine transaminase	76	U/L
Alkaline phosphatase	304	U/L
γ -Glutamyl transpeptidase	50	U/L
Lactate dehydrogenase	185	U/L
Na	137	mEq/L
K	4.4	mEq/L
Cl	102	mEq/L
C-reactive protein	17.9	mg/dL
HbA1c	5.0	%
<Infection>		
β-D glucan	< 6	pg/ml
*Aspergillus* Antigen	Negative	
*C.neoformans* Antigen	Negative	
Anti-HIV Ab	Negative	
<Tumor marker>		
CEA	1.8	ng/mL
SCC	1.2	ng/mL
CYFRA	1.7	ng/mL
NSE	8.1	ng/mL

**Fig. 3:**
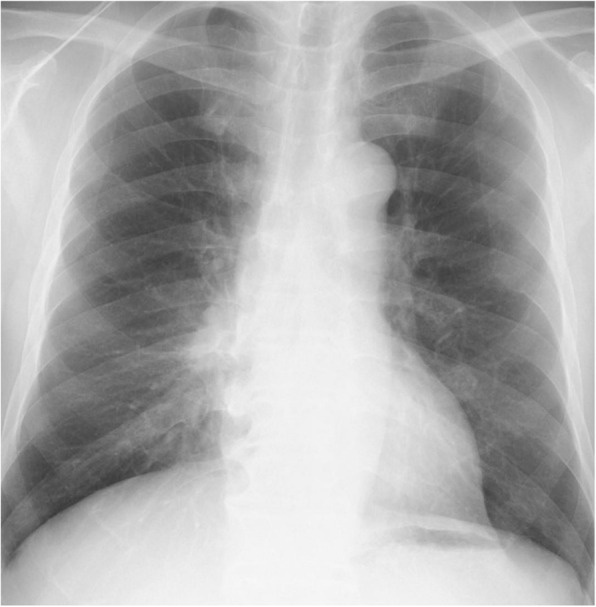
Chest X-ray on 1st discharge at the hospital. Consolidation in the right lower lung field and right pleural effusion were almost disappeared

**Fig. 4:**
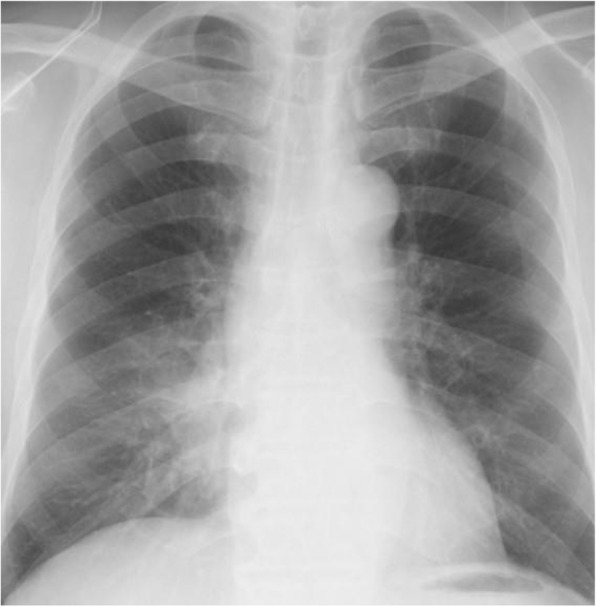
Chest X-ray on 2nd admission at the hospital. Consolidation in the right lower lung field was relapsed

Administration of piperacillin/tazobactam 4.5 g q6hr improved the symptoms. On day 12 from admission, his right pleural effusion disappeared on chest x-ray (Fig. 3), and CRP decreased to 0.17 mg/dL. We switched antibiotic piperacillin/tazobactam to amoxicillin/clavulanate, and he was discharged on Day 14 without fever. After 2 weeks, the patient became febrile, and CRP was elevated to 6.35 mg/dL again. His lung abscess relapsed despite of intaking amoxicillin/clavulanate after he was discharged (Fig. 4). He was admitted again.

**Fig. 5:**
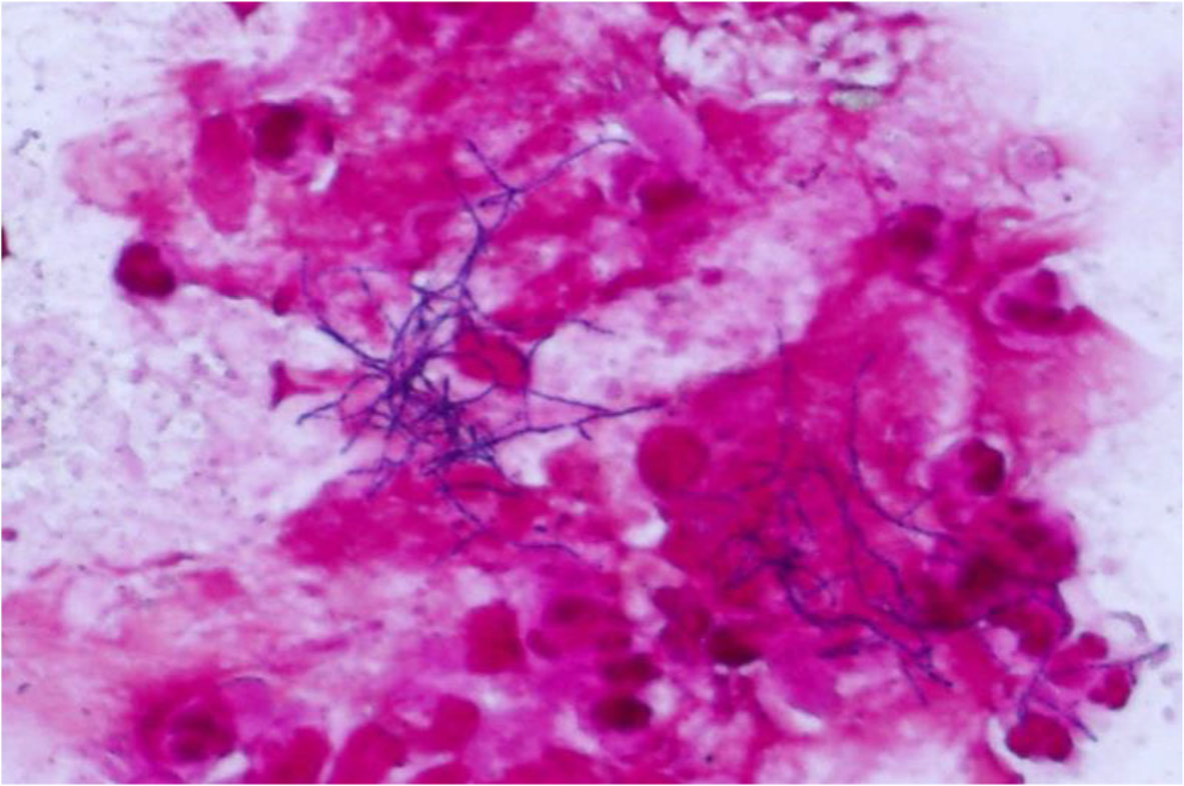
Gram stain (× 1000) of bronchial lavage fluid showing gram-positive, branching, beaded filaments

We decided to commence re-administration of piperacillin/tazobactam 4.5 g q6hr for 3 weeks. Despite getting his symptoms and clinical findings to recover in just 1 week, chest CT findings on day 21 at second admission showed little improvement. We decided to perform bronchoscopy. Bronchial washing and transbronchial lung biopsy through right B^5b^ were performed. Gram-positive, filamentous, branching rods were found by Gram stain (Fig. 5), and negative examination of Ziehl-Neelsen stain. *Nocardia* species were cultured from samples of the bronchial wash.

After we confirm by brain CT scan that no brain abscess existed, we switched to TMP/SMX. He was an immunocompetent patient, and he did not have a CNS problem. Susceptibility testing was performed according to Clinical and Laboratory Standards Institute (CLSI) document M24-A; the specific susceptible breakpoint of TMP/SMX for Nocardia species was ≤2/38 and the specific resistance breakpoint was ≥4/76. The MICs of TMP/SMX for *N. exalbida* were 0.25/4.75. Thus, the *N. exalbida* was regarded as sensitive to TMP/SMX (Table 2). We decided to prescribe TMP/SMX 2 tablets BID (approximately 5 mg (per kg of body weight) for the TMP component). Treatment response was good, but renal dysfunction occurred from administration of TMP/SMX 4 months later. We switched to minocycline 100 mg po BID for 1 month. After treatment, we confirmed that consolidation on CT had disappeared, and he has not relapsed. With the corporation of the Medical Mycology Research Center of Chiba University, the isolates were identified as *N. exalbida* using 16S rRNA gene sequencing.

**Table 2:** Antimicrobial susceptibilities of *Nocardia exalbida* isolated from Brochial wash fulid cultures

Minimum inhibitory concentration (μg/mL)	
Ampicillin	> 8
Ceftriaxone	4
Imipenem	1
Minocycline	2
Trimethoprim/Sulfamethoxazole	0.2/4.75
Amikacin	< 4
Gentamicin	< 0.5
Ciprofloxacin	4
Clarithromycin	4
Erythromycin	> 2

## Discussion and conclusion

Pulmonary nocardiosis mainly occurs as an opportunistic infection in immunocompromised patients, particularly in those with defect in cell-mediated immunity such as patients with human immunodeficiency virus (HIV) infection and those receiving long-term systemic steroids or immunosuppressive agents. Pulmonary nocardiosis often occurs with chronic obstructive pulmonary disease (COPD) and bronchiectasis [7]. We cannot point out any underlying disease that cause an immunocompromised state (ex. human immunodeficiency virus infection, diabetes) and no malignant disease has developed in the follow up period.

There is no report that discussed the infection route in the past *N. exalbida* case reports. But other pulmonary nocardiosis was often described from environmental history. In our case he was doing gardening as a hobby and might have inhaled from soil.

Susceptibility differs among the species [2]. TMP-SMX is active against most *Nocardia* species; however, *N. otitidiscaviarum* is commonly resistant to TMP-SMX, and *N.nova* and *N. farcinica* are occasionally resistant [8]. So, it is important to identify of *Nocardia* species and determine antimicrobial susceptibility.

*N. exalbida* was first isolated from two immunocompromised patients with a cutaneous lesion and lung abscess in 2006 [9]. To date,10 cases of nocardiosis caused by *N. exalbida* have been reported (Table 3).

**Table:** Table 3   Literature review of *Nocardia exalbida* infection

Reference	Age /sex	Disease	Underlying condition	Treatment	Duration for Antibiotics	Outcome
[9]	43/NR	Pneumonia	NR	NR	NR	NR
[9]	60/NR	NR	Pemphigus -vulgaris	NR	NR	NR
[10]	38/F	Keratitis	None	Erytromycin + topical agent →TMP/SMX(3 tablets/day) +topical agent	unclear 10 days unclear	Improved
[11]	63/M	Brain abscess	Lymphoma	TMP/SMX(13 mg/kg/day) +MEPM(4 g/day) →TMP/SMX(11.5 mg/kg/day)^a^	2 months 2 months > 4 months	Improved
[12]	47/M	Pneumonia	HIV,DM,HB	IMP(2 g/day) AMK(15 mg/kg/day) →GRNX(400 mg/day)^a^	17 days 17 days 6 months^a^	Improved
[13]	56/M	Endophthalmitis	None	TMP/SMX (unknown →Enucleation	6 months	Improved
[14]	68/M	Pneumonia	HIV	TMP/SMX(12 mg/kg/day) →TMP/SMX(6 mg/kg/day)	7 days 12 months	Improved
[15]	57/M	Blebitis	Open-angle glaucoma	TMP/SMX (unknown)		Improved
				+AMK (unknown)	6 months	
				+Sulfonamide (unknown)		
[16]	70/M	Pneumonia	Lung cancer oral steroids	DRPM(3.0 g/day) +TMP/SMX(13 mg/kg/day)	17 days 8 days	Improved ^b^
[17]	76/M	Pneumonia	Colon cancer	TMP/SMX (unknown) →TMP-SMX + LVFX	3 months	Improved
Present case	77/M	Pneumonia	None	TMP/SMX(5 mg/kg/day) →Minocycline 200 mg	4 months 1 month	Improved

^a^maintenance therapy

^b^Nocardiosis was improved, however the patient was died for Lung cancer

Only 6 cases of pulmonary nocardiosis were reported including our case [10, 12, 14], one each with brain abscess [11], keratitis [10], endophthalmitis [13], blebitis [15], and pemphigus vulgaris [9].

All the former cases of pulmonary nocardiosis occurred in immunocompromised patients. Our case is the first reported case of pulmonary nocardiosis by *N. exalbida* in immunocompetent patient.

In the outcomes of all these cases, death by nocardiosis or poor response to treatment for *N. exalbida* infection had not been reported (including two cases where the outcome is unknown and one case is died due to progressive lung cancer).

*N. exalbida* infection might be associated with a good response to treatment [16]. In one case that had pneumonia with *N. exalbida* with HIV the patient was treated with TMP/SMX six tablet (approximately 12 mg per kg of body weight) for the TMP component) monotherapy for 12 month and cure [14]. In our case we prescribed TMP/SMX 4 tablets (approximately 5 mg per kg of body weight) for the TMP component) monotherapy for 4 months and switched to minocycline for 1 month because renal injury induced by TMP/SMX had been suspected. In our case, the response to treatment was good. It was considered that a dose reduction of TMP/SMX in case of *N. exalbida* infection without CNS lesion might occur in an immunocompetent patient.

In general, long-term treatment is recommended for patients with CNS lesions (ex. Brain abscess) or severe immunodeficiency, but there is no consensus on *N. exalbida* even in immunocompetent patient. We considered the disappearance of the abscess as a goal of treatment.

In this case, a diagnosis was delayed. We could not assume pulmonary nocardiosis because our patient did not have an underlying disease except for dyslipidemia and clinically improved immediately following treatment with piperacillin/tazobactam on 1st admission. We could determine causative microorganism and antimicrobial susceptibility performing bronchoscopy on 2nd admission.

Although the symptoms improved with the initial treatment, the abscess remained even after the inflammation improved, so it is probable that he was infected with Nocardia at the time of initial hospitalization.

In conclusion, we reported a case of pulmonary nocardiosis caused by *N. exalbida* in an immunocompetent patient. *N. exalbida* infection might be associated with a good response to treatment. We expect a further accumulation of the clinical characteristics of *N. exalbida*.

## Data Availability

All data discussed in the manuscript are included within this published article.

## Abbreviations

CT: computed tomography
16S rRNA: 16S ribosomal RNA
TMP/SMX: Trimethoprim/sulfamethoxazole
CRP: C-reactive protein
GCS: Glasgow Coma Scale
